# Validity and reliability of the left ventricular assist device self-care behaviour scale

**DOI:** 10.1371/journal.pone.0275465

**Published:** 2023-02-10

**Authors:** Naoko P. Kato, Semyon Melnikov, Quin E. Denfeld, Jesus Casida, Anna Strömberg, Tuvia Ben-Gal, Christopher S. Lee, Tiny Jaarsma

**Affiliations:** 1 Department of Health, Division of Nursing Sciences and Reproductive Health, Medicine and Caring Sciences, Linköping University, Linköping, Sweden; 2 Department of Therapeutic Strategy for Heart Failure, The University of Tokyo Hospital, Tokyo, Japan; 3 Sackler Faculty of Medicine, Department of Nursing, Stanley Steyer School of Health Professions, Tel Aviv University, Tel Aviv, Israel; 4 School of Nursing & Knight Cardiovascular Institute, Oregon Health and Science University, Portland, Oregon, United States of America; 5 Eleanor Mann School of Nursing, The University of Arkansas, Fayetteville, Arkansas, United States of America; 6 Sackler Faculty of Medicine, Heart Failure Unit, Cardiology Department, Rabin Medical Center, Petah Tikva, Tel Aviv University, Tel Aviv, Israel; 7 The Boston College William F. Connell School of Nursing, Chestnut Hill, Massachusetts, United States of America; 8 Mary MacKillop Institute for Health Research, Australian Catholic University, Melbourne, Australia; University of Florida, UNITED STATES

## Abstract

**Background:**

Adequate self-care behaviour is essential for patients with a left ventricular assist device (LVAD) to prevent complications, prolong life, and optimise quality of life. However, there were no valid and reliable measurements available to assess self-care behaviour among patients with LVAD. We have previously developed the 33-item LVAD self-care behaviour scale.

**Objectives:**

To evaluate psychometric properties of the 33-item LVAD self-care behaviour scale.

**Methods and results:**

Data on 127 patients with a LVAD in Israel, Japan, and the USA were analysed (mean age 51±14.3, 81% male). Exploratory factor analysis extracted three factors, and 13 items were excluded from the scale. Internal consistency assessed by Cronbach’s alpha was acceptable for the total scale (α = 0.80) and the three subscales: Factor 1: Monitoring (α = 0.81), Factor 2: Heart failure self-care (α = 0.67), and Factor 3: LVAD self-care (α = 0.63). The 20-item version of the LVAD self-care behaviour scale had sufficient convergent validity with another scale that assessed self-care related to the driveline of LVAD (r = 0.47, p<0.001). Test–retest reliability was adequate (intraclass correlation coefficient = 0.58).

**Conclusions:**

The 20-item version of the LVAD self-care behaviour scale showed adequate validity and reliability. The scale is ready for use in clinical practice and research. Additional testing might further optimise the scale.

## Introduction

With the growing number of heart failure (HF) patients and improved medical management, the prevalence of patients with advanced HF is increasing [1]. The gold standard treatment of patients with advanced HF is heart transplantation, but the supply of donor organs is limited. During the past two decades, mechanical circulatory support, primarily using a left ventricular assist device (LVAD), has emerged as a major alternative treatment option for patients with advanced HF. LVAD devices have been increasingly used worldwide, not only as a bridge to heart transplantation, but also as a destination therapy [2]. More than 22 000 devices have been implanted to date in the USA, and at least 2500 new implants occur annually [3]. Many studies have shown that LVAD treatment improves the patients´ prognosis and quality of life [4,5].

Successful long-term LVAD support requires a high degree of self-care by the patients and their caregivers [6,7]. For example, LVAD-supported patients need to take care of the driveline exit site, monitor for signs of infection as well as worsening heart failure, and respond appropriately to emergency situations. Good self-care at home for LVAD-supported patients may be associated with good prognosis and better quality of life because it may decrease the risk of infection and other LVAD-related complications [8–12]. To identify possible deficits in LVAD self-care behaviour and evaluate the effectiveness of educational support to improve self-care, the patient’s self-care behaviour needs to be measured appropriately using a valid and reliable instrument.

There was only one instrument to measure self-care behaviour in patients after LVAD implantation. This LVAD Patient Home Management Adherence Scale consists of 9 LVAD-specific self-care behaviours, related to the equipment and driveline [13]. However, patients also need to perform more self-care behaviours, such as daily weight monitoring, regular exercise, and fluid intake. A single instrument that could comprehensively measure self-care behaviours would be of great value.

To address this need, we developed a 33-item LVAD self-care behaviour scale based on the method for developing patient-reported outcomes recommended by the US Food and Drug Administration [14,15]. The scale items were generated on the basis of a literature review and 2-round Delphi method with expertise in HF and LVAD, guided by the Middle-Range Theory of Self-care of Chronic Illness [16]. Further refinement is needed to use it in research as well as in clinical practice. The purpose of the study was therefore to assess validity and reliability of the 33-item version of the LVAD self-care behaviour scale.

## Method

### 1. Study design

A cross-sectional survey using a self-administered questionnaire was performed in Japan, Israel, and the United States. Measurement properties of the LVAD self-care behaviour scale were evaluated in reference to the COSMIN Study Design Checklist [17]. Construct validity and internal consistency were tested in LVAD patients in Japan, the United States and Israel; meanwhile Israeli patients were not included in the survey for convergent validity because no Hebrew version of the LVAD Patient Home Management Adherence Scale was available. Test-retest reliability was only feasible in Japan due to logistical problems in other two countries (e.g., lack of research staff, COVID-19). This study was approved by the local ethics committee (No. 10947-(3) in The University of Tokyo, Japan; No. 0818-18-RMC in Rabin Medical Centre, Petach Tikva, Israel; No. 17186 in Oregon Health and Science University, the USA) and informed consent was obtained from all study participants. This study conforms to the principles of the Declaration of Helsinki.

### 2. Study participants

Eligible participants in the present study were at least 20 years old, had received continuous-flow implantable LVAD therapy, and were able to read local languages (i.e., Japanese, Hebrew, or English). Patients were excluded if they had major cognitive impairment; were physically unable to complete the questionnaire as judged by outpatient clinic nurses or other health care professionals; were unable or unwilling to give informed consent; or did not live at home (e.g., lived at a nursing home, hospital). Study participants who met the above criteria were recruited from three university hospitals in Japan, Israel, and the USA between 2016 and 2021. Research nurses provided the patients with an explanation of the purpose of the study, the way the research would be carried out, and a guarantee of their anonymity and data confidentiality. Patients who provided written informed consent were given a questionnaire to complete at the hospital or at home. The questionnaire included the 33-item version of the LVAD self-care behaviour scale, the LVAD Patient Home Management Adherence Scale for the assessment of the convergent validity, and demographic characteristics of the patients. For the evaluation of the test–retest reliability, a questionnaire including a LVAD self-care behaviour scale was sent by mail to the patients 2–3 weeks after the first survey, and the patients were asked to complete it again. Clinical characteristics of the subjects were collected from patients’ medical records by research nurses.

### 3. Measurements

#### 3.1 The 33-item version of an instrument for measuring LVAD self-care behaviour

The 33-item version of an instrument for measuring self-care behaviour after LVAD implantation was developed on the basis of a literature review including qualitative studies, quantitative studies, and LVAD guidelines, as well as a 2-round Delphi method involving 17 clinicians with expertise in heart failure and LVAD from 5 countries [9,15,18]. The middle-range theory of self-care of chronic illness was used as the fundamental theory of the scale [16]. The theory has three key concepts: *self-care maintenance*, *self-care monitoring*, and *self-care management*. In the 33-item LVAD self-care behaviour scale, 19 items assess LVAD self-care maintenance behaviour, which measures activities related to the maintenance of the device and lifestyle. Ten self-care monitoring items address the monitoring of the LVAD driveline, infection, and HF symptoms. Four items measure self-care management, which includes handling alarms and coping with living with the device. Patients were asked how often they performed the behaviour on a 5-point Likert scale from 1 (Never) to 5 (Always). The total score was calculated by the sum of each item score, with higher scores representing more engagement in self-care behaviour.

Since the LVAD self-care behaviour scale was developed in English, the scale was forward-translated into Japanese by two independent Japanese professional translators who were proficient in English, and then back-translated into English by two independent English translators who were proficient in Japanese, according to the standard translation process of the scale [19]. A similar procedure was used for Hebrew, with a forward Hebrew translation and back translation.

Because we developed the 33-item version of the LVAD self-care behaviour scale [15] and we were the owner of the original scale, we did not have to obtain the permission to modify it.

#### 3.2. LVAD patient home management adherence scale

To assess the convergent validity of the LVAD self-care behaviour scale, the LVAD Patient Home Management Adherence Scale developed by Casida et al. [20] was used. For the usage of the scale in Japan, forward and back-translations from the English version were performed in Japan according to the standard translation process of the scale [19]. The scale consists of nine elements of LVAD-specific self-care behaviour, related to the equipment and driveline [13]. The total score is obtained by adding the scores for all 9 items and ranges from 0 to 45. A higher score indicates better adherence of LVAD-specific self-care behaviour. The psychometric properties of the scale have been established [20].

#### 3.3 Patient’s characteristics

The following variables were assessed by the self-administrated questionnaire or medical records: age, gender, marital status, education level, employment status, types of LVAD, duration of LVAD, indications for LVAD therapy, cause of HF, and comorbid conditions such as kidney disease or chronic obstructive pulmonary disease. Body Mass Index and smoking status were also assessed.

### 4. Analysis for psychometric properties

#### 4.1. Item analysis

The means, standard deviations, median, skewness, and kurtosis were explored to assess the distribution of the instrument’s responses. The floor and ceiling effects were assessed by the percentages of patients who obtained the minimum and maximum possible score, respectively. The presence of missing items and item-total correlations was examined.

#### 4.2. Construct validity

Based on the instrument’s theoretical perspective [15], we hypothesised a three-factor model of the LVAD self-care behaviour scale consisting of self-care maintenance, self-care monitoring, and self-care management. To test how well the measured variables fit the theory-derived model, confirmatory factor analysis was performed. To assess model fit, the relative chi-square (CMIN/DF, i.e. the chi-square/degree of freedom), root mean square error of approximation (RMSEA), comparative fit index (CFI), Tucker-Lewis index (TLI), and normed fit index (NFI) were calculated. Thresholds for acceptable fit were the relative chi-square CMIN/DF < 3.0, RMSEA values = 0.05–0.08, CFI and TLI values ≥ 0.95, and NFI values ≥ 0.90 [21–23].

Subsequently, exploratory factor analysis with promax rotation was performed. To determine the number of factors, the Kaiser criterion that eigenvalues be greater than one and the scree plot were used [24]. For factor extraction, a method of principal components was used. When the factor loading of the item was less than 0.30, or the item was cross loaded on multiple factors with a similar magnitude, the decision to keep or remove the items was based on clinical relevance.

#### 4.3. Convergent validity

To assess the convergent validity of the instrument, Pearson´s correlation coefficient was calculated between the total scores of the instrument and the total scores of the LVAD Patient Home Management Adherence Scale [13]. We hypothesised that there would be a moderate or strong correlation (the absolute value of the correlation coefficient being r. ≥ 0.30) between the scales [25], since they were considered to measure similar constructs.

#### 4.4. Reliability

Cronbach’s α and 95% confidences intervals (CI) were calculated as an index of internal consistency. A Cronbach’s α coefficient of 0.70 was considered sufficient [26]. Test-retest reliability was evaluated by the intraclass correlation coefficient (ICC) for the total scale and each subscale used data from the first and second surveys.

#### 4.5. Statistical analysis

Descriptive data are presented as a mean and standard deviation for continuous variables, and nominal scaled variables are displayed as numbers and percentages. Missing items were substituted with a mean score calculated using the rest of the items in cases where up to 50% of the items were missing. All statistical tests were two-tailed, and a *p*-value <0.05 was considered statistically significant. All analyses were performed using SAS version 9.4 for Windows (SAS Institute Inc., Cary, North Carolina, USA) and IBM SPSS Statistics for Windows, version 25.0 (Armonk, NY, USA: IBM Corp).

## Results

### 1. Sociodemographic and clinical characteristics of the participants

Of those approached, 67 of the 84 Japanese patients (80%), 5 of the 9 American patients (56%), and 55 of the 65 Israeli patients (85%) consented and completed the questionnaire. In total, 127 LVAD-supported patients completed the questionnaire (mean age 51years, 81% male). As shown in Table 1, for most of the respondents from Israel and Japan, the indication for transplantation was a bridge to transplant (BTT), while for the patients from the USA, the indication was destination therapy (DT). In total, approximately half of the study patients had HeartMate II, while 20% had HeartMate 3 and 17% had Jarvik2000. The median time patients lived with the LVAD prior to the questionnaire survey was 4 months for Israelis, 13 months for Japanese and 55 months for Americans.

**Table 1:** Sociodemographic and clinical characteristics.

	Total (N = 127)		Israel (N = 55, 43%)		Japan (N = 67, 53%)		USA (N = 5, 4%)	
**Age,** Mean±SD	51.0±14.3		61.9±8.8		41.5±10.9		57.2±15.3	
**Gender**, Male, n (%)	103	(81%)	50	(91%)	51	(76%)	2	(40%)
**Marital status**, n (%) Single Married Divorced Widowed	27 79 18 1	(22%) (63%) (14%) (1%)	3 41 11 0	(6%) (74%) (20%) (0%)	23 36 5 1	(35%) (55%) (8%) (2%)	1 2 2 0	(20%) (40%) (40%) (0%)
**Education**, n (%) Less than high school High school College/university Graduate school Other	9 54 46 15 2	(7%) (43%) (36%) (12%) (2%)	6 26 10 11 1	(11%) (48%) (19%) (20%) (2%)	3 25 34 4 1	(5%) (37%) (51%) (6%) (1%)	0 3 2 0 0	(0%) (60%) (40%) (0%) (0%)
**Employment**, n (%) Employed Unemployed	19 108	(15%) (85%)	5 50	(9%) (91%)	14 53	(21%) (79%)	0 5	(0%) (100%)
**LVAD name**, n (%) HeartWare HVAD HeartMate II HeartMate 3 DuraHeart EVAHEART Jarvik2000	10 60 25 3 7 21	(8%) (47%) (20%) (2%) (6%) (17%)	6 27 22 0 0 0	(11%) (49%) (40%) (0%) (0%) (0%)	1 33 1 3 7 21	(2%) (49%) (2%) (5%) (11%) (31%)	3 0 2 0 0 0	(60%) (0%) (40%) (0%) (0%) (0%)
**Indication**, n (%) Bridge to transplant Destination therapy	114 13	(90%) (10%)	45 10	(82%) (18%)	67 0	(100%) (0%)	2 3	(40%) (60%)
**Cause of HF**, n (%) NICM ICM Other Don’t know	78 36 6 7	(61%) (28%) (5%) (6%)	19 29 2 5	(35%) (53%) (4%) (9%)	58 6 3 0	(87%) (9%) (5%) (0%)	1 1 1 2	(20%) (20%) (20%) (40%)
**Comorbidities**, n (%)								
Diabetes	45	(35%)	32	(58%)	11	(16%)	2	(40%)
Arrhythmia	52	(41%)	33	(60%)	17	(35%)	2	(40%)
Anemia	22	(17%)	19	(35%)	0	(0%)	3	(60%)
Chronic renal failure	39	(31%)	28	(51%)	9	(13%)	2	(40%)
COPD	5	(4%)	4	(7%)	0	(0%)	1	(20%)
**Smoking, yes**, n (%)	3	(2%)	3	(6%)	0	(0%)	0	(0%)
**Time since LVAD implantation (months)** Median (q1-q3)	9.7 (3.9–21.8)		4.1(1.5–16.9)		12.2 (7.0–22.0)		54.7 (7.0–22.0)	
**Time since HF diagnosed (months)** Median (q1-q3)	70.1 (32.1–145.2)		62.5 (27.4–146.3)		72.7 (32.8–146.9)		83.0 (57.8–182.5)	
**Body mass index** Mean±SD	24.4±6.0		28.0±5.2		21.0±3.9		31.7±10.3	

COPD, chronic obstructive pulmonary disease; HF, heart failure; ICM, ischemic cardiomyopathy; LVAD, Left ventricular assist device; NICM, non-ischemic cardiomyopathy.

### 2. Descriptive statistics of the self-care scale items

Table 2 shows the descriptive statistics of the LVAD self-care behaviour scale. Mean scores per item ranged between 3.5 (item 21) and 5.0 (item 33), indicating that patients usually performed most of self-care behaviour tasks. Median scores ranged between 4 and 5 (most of the time and always), indicating the answers were skewed to the left, resulting in a left tail [27]. All estimates of skewness were negative and ranged from -0.54 (item 24) to -7.53 (item 33), indicating a tail to the left for all items; kurtosis ranged from -0.97 (item 21) to 60.67 (item 33). Several items demonstrated low item-total correlation (Pearson’s r <0.2) (items 10, 11, 14, 29, 31, 33), indicating that these items have a weak relationship to other items [28]. All other items had satisfactory item-total correlation ranging between 0.20 and 0.54.

**Table 2:** Descriptive statistics of the 33 items of the LVAD self-care scale.

		N	Mean	SD	Median	Items responses (%)					Skewness	Kurtosis	Range	% missing	Item total correlation	Cronbach’s Alpha if deleted
						1	2	3	4	5						
1	I clean the controller, batteries and battery connection	127	4.2	1.00	4.0	2.4	4.7	15	31.5	46.5	-1.18	0.97	4	<0.01	0.25	0.79
2	I check that the electric and battery power sources are available and work properly	127	4.7	0.59	5.0	0	0.8	4.7	15.7	78.7	-2.26	4.96	3	<0.01	0.29	0.79
3	When I go to sleep, I keep the driveline, controller, and power supply secured	127	4.9	0.41	5.0	0	0.8	0	11.8	87.4	-3.73	18.45	3	<0.01	0.32	0.79
4	I keep both the back up battery and controller with me	127	4.6	0.90	5.0	3.1	0.8	7.1	14.2	74.8	-2.49	6.24	4	<0.01	0.24	0.79
5	I avoid kinking, pulling or moving the driveline at the exit site	127	4.5	0.73	5.0	0.8	0.8	7.0	26.8	64.6	-1.85	4.28	4	<0.01	0.47	0.78
6	I wear a stabilisation device to keep the driveline in place and to avoid excessive movement at the exit site	126	4.8	0.57	5.0	0	1.6	2.4	13.5	82.5	-2.91	9.30	3	0.79	0.42	0.79
7	I keep the exit site and driveline clean and dry	127	4.8	0.60	5.0	0.8	0.8	1.6	16.5	80.3	-3.34	14.46	4	<0.01	0.30	0.79
8	I follow the steps/instructions in changing the dressing on the exit site of the driveline	127	4.9	0.23	5.0	0	0	0	5.5	94.5	-3.95	13.78	1	<0.01	0.28	0.79
9	I check and record the LVAD speed, flow, power and PI	127	4.4	1.14	5.0	5.5	3.9	7.9	11.0	71.7	-1.89	2.52	4	<0.01	0.28	0.79
10	I inspect all cable connectors and the driveline for dirt or damage	126	4.1	1.07	4.0	4.0	4.0	15.9	28.6	47.6	-1.24	1.03	4	0.79	0.14	0.80
11	I monitor my driveline exit site for evidence of infection and drainage as instructed	127	4.7	0.71	5.0	1.6	0.8	3.1	11.8	82.7	-3.41	13.12	4	<0.01	0.19	0.79
12	I monitor myself for signs of infection including fever, chills and night sweats	127	4.7	0.78	5.0	2.4	0.8	2.4	18.1	76.4	-3.07	10.66	4	<0.01	0.41	0.78
13	I monitor myself for any signs of blood in my nose, urine (color change) or blood in my stools	127	4.6	0.87	5.0	3.1	0.8	3.9	17.3	74.8	-2.77	8.08	4	<0.01	0.40	0.78
14	I check my INR at home or clinic as instructed	124	4.8	0.61	5.0	0	1.6	4.8	7.3	86.3	-2.98	8.60	3	2.4	0.09	0.79
15	I contact the LVAD/heart failure team in case of alarms or equipment issues	124	4.6	0.94	5.0	3.2	1.6	7.3	12.9	75.0	-2.38	5.40	4	2.4	0.27	0.79
16	I can talk to someone about coping with the LVAD or my health condition	123	4.7	0.65	5.0	0	0.8	7.3	16.3	75.6	-1.93	3.01	3	3.2	0.34	0.79
17	I monitor myself for the development of or increase in leg swelling	124	4.7	0.67	5.0	1.6	0	2.4	16.9	79.0	-3.40	14.54	4	2.4	0.54	0.78
18	I monitor myself for worsening shortness of breath	124	4.7	0.72	5.0	1.6	0.8	2.4	19.4	75.8	-3.02	11.13	4	2.4	0.45	0.78
19	I monitor myself for worsening fatigue	124	4.7	0.66	5.0	1.6	0	1.6	18.5	78.2	-3.47	15.52	4	2.4	0.44	0.79
20	I weigh myself	124	4.6	0.81	5.0	0.8	2.4	8.1	15.3	73.4	-2.10	4.25	4	2.4	0.43	0.78
21	I talk with a LVAD/heart failure team or someone when I am feeling sad or worried	122	3.5	1.42	4.0	15.6	8.2	21.3	23.0	32.0	-0.54	-0.97	4	3.9	0.20	0.80
22	I adjust my physical activities according to my symptoms	121	4.4	0.86	5.0	1.7	2.5	7.4	28.1	60.3	-1.83	3.64	4	4.7	0.51	0.78
23	I take my medicine as prescribed	123	5.0	0.22	5.0	0	0	0	4.9	95.1	-4.24	16.25	1	3.2	0.33	0.79
24	I perform regular physical activity	124	3.6	1.24	4.0	7.3	13.7	21.0	29.8	28.2	-0.54	-0.70	4	2.4	0.31	0.79
25	I eat a heart healthy diet	124	3.9	0.92	4.0	1.6	2.4	29.0	35.5	31.5	-0.55	0.09	4	2.4	0.45	0.78
26	I follow the daily recommended fluid intake	124	4.3	0.88	4.0	1.6	2.4	11.3	37.1	47.6	-1.36	2.16	4	2.4	0.43	0.78
27	I limit my alcohol intake to 1 unit/day for females and 2 units per day for males	123	4.6	1.20	5.0	8.9	1.6	0.8	1.6	87.0	-2.52	4.61	4	3.2	0.34	0.79
28	I avoid cigarettes and tobacco smoke	124	4.6	1.09	5.0	7.3	0.8	1.6	3.2	87.1	-2.83	6.53	4	2.4	0.36	0.78
29	I get enough sleep	123	4.2	0.90	4.0	0.8	4.1	16.3	35.0	43.9	-0.96	0.49	4	3.2	0.13	0.79
30	I contact the LVAD /heart failure team in case of symptoms	123	4.6	0.87	5.0	1.6	2.4	8.1	14.6	73.2	-2.17	4.57	4	3.2	0.22	0.79
31	I measure my blood pressure	123	3.8	1.60	5.0	17.1	9.8	11.4	4.9	56.9	-0.75	-1.13	4	3.2	0.19	0.80
32	I monitor myself for the symptoms of stroke	120	4.0	1.30	5.0	10.0	3.3	12.5	21.7	52.5	-1.27	0.47	4	5.5	0.34	0.79
33	I come to scheduled clinical visits	126	5.0	0.22	5.0	0	0	0.8	1.6	97.6	-7.53	60.67	2	0.79	0.07	0.79

INR, International normalized ratio; LVAD, Left ventricular assist device; PI, Pulsatility index; SD, standard deviation.

### 3. Construct validity

In the confirmatory factor analysis of the 33-item version of the LVAD self-care behaviour scale, most of fit indices did not reach thresholds of acceptability. The model-fit measures obtained were CMIN/df = 2.445, p-value<0.001, RMSEA = 0.102, CFI = 0.482, NFI = 0.377, TLI = 0.409. Factor loadings for a total of 11 items (seven items in self-care maintenance, two items in self-care monitoring, and three items in self-care management) were lower than 0.30. Thus, the fit of the 33-item LVAD self-care behaviour scale with the three subscales was not considered acceptable.

We therefore proceeded with an exploratory factor analysis. According to the scree plots and the Kaiser criterion that eigenvalues be >1, three factors were extracted from the 33-item version of the LVAD self-care behaviour scale. In accordance with the following three steps, the number of items was reduced from 33 to 20 (Table 3). As Step I, six items were removed: item 8 (follow the instructions about changing the dressing), item 23 (take my medicine), and item 33 (attend scheduled clinical visits) due to not being discriminating (Mean, 4.9 and above, Median 5). Item 31 (measure my blood pressure) was excluded because not all patients had a device with which to measure blood pressure or were not able to measure blood pressure by themselves. Item 27 (limit my alcohol intake) and item 28 (avoid cigarettes) were excluded, since they describe general self-care. In addition, adherence of the two self-care behaviours were relatively high (approximately 90%), which means that these items could not discriminate the patients sufficiently. Moreover, we observed that 3.2% of patients were missing data on the alcohol item, which might be reflected the difficulty in answering the items. In the scale, patients were asked if they limited alcohol intake to one unit/day for females and two unit/day for males and the information on alcohol units was provided (e.g., a bottle of beer 500 ml). Because there are many types of alcoholic beverages in the world, patients should be asked alcohol intake with sufficient information separately from the scale. For these reasons, we removed the item from the scale. After excluding 6 items, a new exploratory factor analysis of 27 items was performed (Step II). Two items with factor loading <0.3 were excluded: item 1 (clean the controller and batteries) and item 4 (keep both the back up battery and controller with me). Subsequently, after an exploratory factor analysis of 25 items (Step III) the following five items were excluded: item 9 (check and record the LVAD speed) due to negative loading and its clinical irrelevance to new device design; and item 6 (wear a stabilisation device) since it is not relevant for self-care at home. After discussion among the research team, item 30 (contact the LVAD /heart failure team in case of symptoms) and item 21 (talk with the LVAD/heart failure team or someone when I am feeling sad or worried) were excluded because of their redundancy. Item 10 (I inspect all cable connectors and the driveline for dirt or damage) overlapped with item 7 (I keep the exit site and the driveline clean and dry), making the weight of the cable inspections too heavy in the scale; hence item 10 was removed. As Step IV, an exploratory factor analysis of 20 items was performed. The results showed that the data set was adequate [(KMO) coefficient = .79, Bartlett Sphericity Test (χ2) = χ2 = 834.8, p < .000)]. Three factors explained 46.7% of the total variance of the scale, and the 20 items were classified into three subscales, based on factor loading and clinical relevance, as follows (Table 4): Factor 1 Monitoring, Factor 2 HF self-care, and Factor 3 LVAD self-care.

**Table 3:** Item reductions with reasons.

**Step I**	**Descriptive analysis**	**Reasons**
	Item 8: I follow the steps when changing the dressing Item 23: I take my medicine as prescribed Item 33: I come to scheduled clinical visits	Three items had high mean values of ≥4.9, a median value of 5.0, and a score range of 1 to 2, thus these items could not discriminate patients.
	Item 31: I measure my blood pressure daily	Not all patients had a blood pressure measuring device/were able to measure blood pressure on their own.
	Item 27: I limit my alcohol intake Item 28: I avoid cigarettes and tobacco smoke	They are general self-care.
**Step II**	**Exploratory factor Analysis on 27 items**	
	Item 1: I clean the controller, batteries and battery connection Item 4: I keep both the back up battery and controller with me	Factor loading <0.3
**Step III**	**Exploratory factor Analysis on 25 items**	
	Item 9: I check and record the LVAD speed, flow, power and PI	Negative loading and clinical irrelevance to new devices design.
	Item 6: I wear a stabilisation device to keep the driveline in place	Not relevant at home.
	Item 30: I contact the LVAD team in case of symptoms	There is an item about contacting the team in case of alarms.
	Item 21: I talk with LVAD team or someone when I am feeling sad Item 10: I inspect all cable connectors and the driveline for dirt	Redundancy
**Step IV**	**Exploratory factor analysis of 20 items**	

**Table 4:** Results of exploratory factor analysis using Promax rotation: 20-item LVAD self-care behaviour scale (N = 127).

		Items	Factor loadings			Cronbach’s alpha if item deleted
			Factor 1	Factor 2	Factor 3	
	**Factor 1: Monitoring** (Mean±SD, 87.6±17.5, Cronbach’s alpha 0.81)					
12	I monitor myself for signs of infection including fever, chills and night sweats		**0.870**	0.297	0.300	0.751
18	I monitor myself for worsening shortness of breath		**0.864**	0.186	0.248	0.750
19	I monitor myself for worsening fatigue		**0.851**	0.159	0.312	0.762
13	I monitor myself for any signs of blood in my nose, urine or blood in my stools.		**0.816**	0.241	0.327	0.754
11	I monitor my driveline exit site for evidence of infection and drainage as instructed.		**0.541**	0.241	-0.151	0.801
32	I monitor myself for the symptoms of stroke		**0.505**	0.017	0.244	0.833
20	I weigh myself		**0.484**	0.233	-0.061	0.805
17	I monitor myself for the development of or increase in leg swelling		**0.413**	0.683	0.207	0.809
	**Factor 2: Heart failure self-care** (Mean±SD, 76.2±16.1, Cronbach’s alpha 0.67)					
22	I adjust my physical activities according to my symptoms		0.333	**0.743**	0.246	0.598
25	I eat a heart healthy diet		0.134	**0.717**	0.334	0.560
26	I follow the daily recommended fluid intake		0.105	**0.701**	0.352	0.587
24	I perform regular exercise		0.096	**0.578**	-0.099	0.669
29	I get enough sleep		-0.006	**0.395**	0.200	0.666
	**Factor 3: LVAD self-care** (Mean±SD, 91.1±11.0, Cronbach’s alpha 0.63)					
7	I keep the exit site and driveline clean and dry		0.193	0.305	**0.776**	0.529
2	I check that the electric and battery power sources are available and work properly		0.167	0.212	**0.772**	0.559
3	When I go to sleep, I keep the driveline, controller, and power supply secured		0.329	0.230	**0.613**	0.591
16	I can talk to someone about coping with the LVAD or my health condition		0.212	0.443	**0.549**	0.572
15	I contact the LVAD/heart failure team in case of alarms or equipment issues		0.276	0.037	**0.417**	0.658
14	I check my INR at home or clinic as instructed		-0.005	0.187	**0.362**	0.645
5	I avoid kinking, pulling or moving the driveline at the exit site		0.237	0.456	**0.334**	0.617
	**Total score** (Mean±SD, 85.4±14.0, Cronbach’s alpha 0.80 (95% CI, 0.75, 0.85)					

All scores range from 0 to 100 with higher scores representing more engagement in self-care behaviour.

Abbreviation: HF, heart failure; LVAD, left ventricular assist device; SD, standard deviation.

### 4. Scoring

Scores for the total scale and each subscale were calculated by the sum of each item score with the score range standardised between 0 and 100 as follows below. Higher scores represent more engagement in self-care behaviour. An example of the scoring is provided in S1 Fig.


$$z_{i}=\frac{Sum\;of\;scores\;(xi)-minium\;possible\;score}{\mathrm{Maximum}\;\mathrm{possible}\;\mathrm{score}-minumum\;possible\;score}*100$$

- z_i_: The i^th^ standardised value
- x_i_: The i^th^ value in the dataset

In this sample, the total mean score of the newly developed 20-item LVAD self-care behaviour scale was 85.4±14.0. The mean scores of the subscales were 87.6±17.5, 76.2±16.1 and 91.1±11.0 for Monitoring, HF self-care and LVAD self-care, respectively. The ceiling effect in which the total score of 100 was observed in 4 out of 127 (3%) patients, whereas no floor effect was observed.

### 5. Convergent validity

For the convergent validity, data from LVAD supported patients in Japan and the USA were analysed (n = 72). The total score of the LVAD Patient Home Management Adherence Scale was 40.8 ±4.0. The mean scores of the total LVAD self-care behaviour scale and subscales among the 72 patients were 87.8 ±9.5, 92.1±10.2 (monitoring), and 79.3±13.5 (HF self-care), and 92.1±10.2 (LVAD self-care).

There were moderate correlations between the LVAD Patient Home Management Adherence Scale and both the overall LVAD self-care behaviour scale (r = 0.47, p<0.001) and LVAD self-care behaviour subscale of HF self-care (r = 0.46, p<0.001). On the other hand, we observed small, but significant, correlations between the LVAD Patient Home Management Adherence Scale and both the monitoring (r = 0.28, p = 0.016) and LVAD self-care subscales (r = 0.28, p = 0.016).

### 6. Internal consistency

The Cronbach’s alpha value of the LVAD self-care behaviour scale’s total score was 0.80 (95% CI = 0.75–0.85) (Table 4), and the following Cronbach’s alpha for the following subscales were obtained: 0.81 (Factor 1. Monitoring), 0.67 (Factor 2. HF self-care), and 0.63 (Factor 3. LVAD self-care), respectively.

### 7. Test-retest reliability

In total, data on 18 Japanese patients were analysed. Mean age was 42±12 years old, and 67% (n = 12) were male. The ICC of the LVAD self-care behaviour scale’s total score was 0.58 (Table 5), while the ICC of the subscales ranged between 0.51 and 0.55. Of the 18 patients, five patients received some additional training between the first and second survey (e.g., use new gloves when sterilising the drive line, get enough fluids, learn how to handle emergency situations, learn how to come back to work). When removing these five patients from the analysis, the ICC was 0.50.

**Table 5:** Test-retest reliability of the 20-item LVAD self-care behaviour scale (n = 18).

	The first survey	The second survey	Intraclass correlation coefficient
	Mean score ±SD	Mean score ±SD	
Total score	90.1±7.0	91.9±10.0	0.58
Scores of subscales			
Monitoring	94.0±7.2	94.6±11.4	0.51
Heart failure self-care	81.7±7.2	86.3±10.4	0.55
LVAD self-care	94.0±7.2	94.6±11.4	0.51

All scores range from 0 to 100 with higher scores representing more engagement in self-care behaviour.

Abbreviation: LVAD, left ventricular assist device; SD, standard deviation.

A copy of the 20-item LVAD self-care behaviour scale is provided in S2 Fig.

## Discussion

In the present study, we performed initial psychometric testing and further refinement of the LVAD self-care behaviour scale. We previously developed a 33-item scale based on a qualitative and quantitative literature review, guidelines and discussions among the research team (including clinical experts on heart failure and LVAD [15]), and the Middle Range Theory of Self-Care of Chronic Illness [16]. We demonstrated that the 20-item LVAD self-care behaviour scale is a valid and reliable instrument to measure self-care behaviour for LVAD supported patients in research and clinical practice.

We did not confirm the three established constructs of the Middle Range Theory of Self-Care (i.e., self-care maintenance, self-care monitoring, self-care management) in this context of LVAD implantation and self-care [16]. Using an exploratory factor analysis, we derived a clear factor that was focused on monitoring that spans both LVAD-specific and general HF-related behaviour, which is similar to the Middle Range Theory of Self-Care [16]. We also identified, however, two new factors related to self-care after LVAD implantation. First, HF-specific self-care behaviour was identified, which spans the continuum from pre-implantation to the post-LVAD phase. Of particular note, the HF self-care behaviour factor we identified in this sample only focuses on what would be identified as self-care maintenance in other illness contexts [29]. Second, a factor focused on LVAD-specific self-care behaviour was identified, but this only relates to device-related care. Interestingly, the LVAD self-care behaviour we identified consists of what is described as a mix of self-care maintenance and self-care management behaviour in other illness contexts [29]. This new way of parsing out underlying illness (HF) and treatment-specific self-care (LVAD) reflects the patient perspective that LVAD self-care behaviour is additional self-care behaviour that HF patients need to perform.

It is also important to note that most scores of each item had a left-skewed distribution, which means most of the patients reported good self-care in the study. This may be partly associated with higher levels of LVAD care self-efficacy, particularly around 6 months after the implantation, given that the median duration of LVAD support in the study population is nearly 9.7 months [30]. Furthermore, this might be attributed to the three institutions where study patients were recruited. The three institutions were one of the major university hospitals regarding LVAD implantations in each country, therefore experienced healthcare professionals provided high-quality LVAD nursing care pre- and post LVAD surgery. For example, prior to discharge at each institution LVAD patients and their informal caregivers received necessary education on the following: LVAD equipment, wound management, driveline´s care, general self-care (e.g., medication, diet, physical activity), daily monitoring of physical status (e.g., blood pressure, weight, pulse) and signs of LVAD complications. After the discharge, a multidisciplinary LVAD team including VAD nurses provided the patients with self-care support regularly. The contents of education were decided according to the national and international guidelines about LVAD management [9,31,32].

Considering the higher rate of LVAD complications due to a lack of self-care behaviour [33], further study is expected to examine which items of the scale are associated with subsequent LVAD complications, as well as assess predictive validity and create a cut-off value for the LVAD self-care behaviour scale, which would identify the patients at higher risk of adverse outcomes.

During the process of factor analysis, several items were removed from the scale because of inadequate psychometric properties. The result is a 20-item scale that can be used in research studies (e.g., evaluating the effect of educational interventions). However, clinicians might be interested in the removed items since they cover different types of clinically important self-care behaviour. For daily clinical practice, the 33-item version of the LVAD self-care behaviour scale can be used, for example, as a checklist for education of patients and caregivers. In the present study, we created a score ranging from 0 to 100, which makes it possible to easily understand the result. Even if the number of items in the scale is changed after further psychometric testing, the current score can be compared with the future score.

Convergent validity was considered adequate when we examined the relationship between the 20-item LVAD self-care behaviour score and LVAD patient home management adherence scale [20]; nevertheless, the correlation was not strong. Most items on the LVAD patient home management adherence scale [20] focus on the LVAD driveline, while our LVAD self-care behaviour scale encompasses comprehensive LVAD self-care behaviour and HF self-care behaviour. These differences may influence the strength of the correlation.

When we examined the Cronbach’s alpha value on the total LVAD self-care behaviour scale, the value was higher than the recommended value of 0.70 [34]; moreover, reliability assessed by internal consistency was considered adequate. Approximately two thirds of the items had an item-total correlation of ≥0.30. These results suggest a single factor model for the 20-item LVAD self-care behaviour scale, and it allows one to calculate a summary score using all 20 items.

The ICC of the LVAD self-care behaviour scale total score was 0.58. According to Koo et al. [35], the total score of the scale had moderate reliability. The reasons why the ICC value did not reach a level of good reliability (the value of 0.75 or higher) might be partly explained by that the first survey might have educational effects, causing some patients to change their behaviour in the second survey. Future studies are needed to confirm test-retest reliability in a larger sample.

### Study limitations and further research

We acknowledge several limitations of the study. Firstly, since the sample size was relatively small, the results may not necessarily be representative, and we could not describe the results of the LVAD self-care behaviour scale by geographic region. Furthermore, convergent validity and test-retest reliability of the scale were not tested in the full sample of the patients. Compared with other more common HF therapies (e.g., diuretics, beta-blockers), LVADs are implanted less frequently, which can make it difficult to obtain large sample sizes. We attempted to mitigate this problem by recruiting patients from multiple geographic regions. Moreover, this study was a preliminary psychometric analysis, and further research will be needed.

Secondly, LVAD self-care was generally quite high in this sample and may not represent the self-care of the general population of patients with implanted LVAD. In particular, most patients received an LVAD as BTT (90%) and had only been living with a LVAD for a short time. Almost half of the participants were recruited from Japan when only the indication for BTT was available. Since LVAD-DT was approved and reimbursed in May 2021 in Japan, the number of LVAD-DT patients will increase in Japan [36] as well as other Western counties [37]. Adherence levels of self-care might be different according to the LVAD implantation strategy, e.g., BTT and DT. Further testing of the validity and reliability of the scale is necessary in a larger sample and should include a more diverse study population.

## Conclusion

After the initial testing of the psychometric evaluation of the LVAD self-care behaviour scale in the present study, the 20-item LVAD self-care behaviour scale is ready for use with LVAD-supported patients in daily clinical practice as well as for research purposes.

## Supporting information

S1 Fig How to standardise the total score on the 20‐item LVAD self-care behaviour scale. (PDF)

S2 Fig The left ventricular assist device self-care behaviour scale. (PDF)
