# Supplementary material for: Validity and reliability of the left ventricular assist device self-care behaviour scale
Source: PLoS One. 2023 Feb 10;18(2):e0275465. doi: 10.1371/journal.pone.0275465 (PMC9917258; doi:10.1371/journal.pone.0275465)
Supplement: S1 Fig — (PDF) [file pone.0275465.s001.pdf]

To standardise the total score on the 20-item LVAD self-care behaviour scale:

1. Sum the responses of the 20 items then subtract the lowest possible scale score of 20.
2. Determine the possible range of scores based on the number of scale items. For the 20-item total scale, scores can range from a high of 100 to a low of 20. Subtract the lowest possible scale score from the highest possible scale score (100 – 20 = 80).
3. Divide the number obtained by in step one by the number obtained in step three and multiple by 100.
