# Supplementary material for: Validity and reliability of the left ventricular assist device self-care behaviour scale
Source: PLoS One. 2023 Feb 10;18(2):e0275465. doi: 10.1371/journal.pone.0275465 (PMC9917258; doi:10.1371/journal.pone.0275465)
Supplement: S2 Fig — (PDF) [file pone.0275465.s002.pdf]

The left ventricular assist device self-care behaviour scale

This scale contains statements about self-care for patients with a left ventricular assist device (LVAD). Respond to each statement by circling the number you think best applies to you. Note that the different answer alternatives constitute a scale ranging between the extremes of 'Never' (1) and 'Always' (5). Even if you feel uncertain about a particular statement, circle the number you feel is most true for you.

	**Never**	**Rarely**	**Sometimes**	**Frequently**	**Always**
1. I evaluate and monitor my driveline exit site for evidence of infection and drainage as instructed.	1	2	3	4	5
2. I monitor myself for signs of infection including fever, chills and night sweats	1	2	3	4	5
3. I monitor myself for any signs of blood in my nose, urine (color change) or blood in my stools.	1	2	3	4	5
4. I monitor myself for the symptoms of stroke.	1	2	3	4	5
5. I monitor myself for the development of or increase in leg swelling	1	2	3	4	5
6. I monitor myself for worsening shortness of breath	1	2	3	4	5
7. I monitor myself for worsening fatigue	1	2	3	4	5
8. I weigh myself	1	2	3	4	5
9. I adjust my physical activities according to my symptoms	1	2	3	4	5
10. I perform regular exercise	1	2	3	4	5
11. I eat a heart healthy diet	1	2	3	4	5
12. I follow the daily recommended fluid intake	1	2	3	4	5
13. I get enough sleep.	1	2	3	4	5
14. I check that the electric and battery power sources are available and work properly	1	2	3	4	5
15. When I go to sleep, I keep the driveline, controller, and power supply secured	1	2	3	4	5
16. I avoid kinking, pulling or moving the driveline at the exit site	1	2	3	4	5
17. I keep the exit site and driveline clean and dry	1	2	3	4	5
18. I check my INR at home or clinic as instructed	1	2	3	4	5
19. I contact the LVAD/heart failure team in case of alarms or equipment issues	1	2	3	4	5
20. I can talk to someone about coping with the LVAD or my health condition	1	2	3	4	5

This is a 20-item version of the left ventricular assist device (LVAD) self-care behaviour scale. The LVAD self-care behaviour scale consists of three factors: Factor 1 Monitoring (from item 1 to item 8), Factor 2 Heart failure self-care (from item 9 to item 13) and Factor 3 LVAD self-care (item 14 to item 20). Scores for the total scale and each subscale score are calculated by the sum of each item score and then the score range is standardised between 0 and 100 as follows. Higher scores represent having engaged in more self-care behaviour.
